# Transient Global Amnesia in a Patient Presenting with Hypertensive Emergency; a Case Report

**Published:** 2020-07-28

**Authors:** Takafumi Obara, Tsuyosi Nojima, Hitoshi Koga, Atsunori Nakao, Hiromichi Naito

**Affiliations:** 1 Department of Emergency, Critical Care and Disaster Medicine, Okayama University Graduate School of Medicine, Dentistry and Pharmaceutical Sciences, Okayama, Japan.; 2 Department of Emergency Medicine, St. Mary Hospital, Kurume, Fukuoka, Japan.

**Keywords:** Amnesia, transient global, memory disorders, htpertension, arterial pressure, hypertension, stroke, emergencies

## Abstract

Transient global amnesia (TGA) is characterized by the abrupt onset of global amnesia, particularly anterograde amnesia. The pathophysiology of TGA is poorly understood and it could be caused by various factors and be associated with various diseases. We report a 58-year-old man who presented to the local emergency room with TGA lasting for several hours. The patient had complete anterograde amnesia without a past medical history of migraine or neurological findings. His systolic blood pressure on presentation was 220 mmHg, which was immediately treated with intravenous calcium ion influx inhibitor.

Other than global amnesia, there was no evidence of neurological disturbance. Computed tomography and magnetic resonance imaging results were unremarkable. After treatment of his hypertension, his amnesia resolved within 12 hours. Emergency department physicians may encounter TGA. Correct diagnosis of the condition depends on recognizing the disease.

## Introduction

Transient global amnesia (TGA) is characterized by the abrupt onset of global amnesia, particularly anterograde amnesia. It is usually a self-limiting condition and does not coincide with other neurological symptoms or signs. While various causes, such as migraine, focal ischemia, epilepsy, or metabolic aspects, have been proposed, the pathophysiology of TGA has not been fully elucidated. TGA is considered to be a result of multiple causes rather than a single mechanism (1).

We treated the unique case of a patient with a typical history of TGA associated with hypertensive emergencies. There have been only a few reports showing the relationship between hypertension and TGA episodes (1-3). Since TGA presents very dramatically and is sometimes seen in the emergency department, emergency physicians must be familiar with this condition. Sharing our experience may help emergency physicians to successfully diagnose TGA by recognizing its characteristic features and avoid unnecessary testing.

## Case Presentation:

A 58-year-old man came to the emergency department with amnesia since 2 hours before admission. His past history included only hypertension and treatment with amlodipine (5 mg/day). Additionally, he had no family psychiatric history. 

He had woken up normally and had breakfast as usual prior to the onset of his amnesia. His family members noticed his repetitive questioning regarding where he was and how he got there, indicating his inability to encode new memories (“How did I come here?”; *“Why I am here?”)*. On arrival, he was alert and calm. The patient presented no seizures, altered mental status, headache, or visual disturbances. His vital signs were as follows; body temperature 36.7°C, blood pressure 220/118 mmHg, and heart rate 84 beats/minutes. Physical examination was unremarkable. Other than the recent memory disturbance, there were no abnormal neurological signs such as sensory disturbance or muscle weakness. All his higher cortical functions like reasoning, calculations, language, and abstract thinking were intact. On interview, he could not recall why he had come to the hospital; however, he clearly stated his name, his work history, and his address. The patient could identify his family members. His biochemical and hematological indices were within normal limits. Intravenous nicardipine (2 mg) was administered and his systolic blood pressure (SBP) dropped to 140-150 mmHg. Brain computed tomography (CT) scan demonstrated no hemorrhage or space occupied by a brain lesion. Magnetic resonance imaging (MRI) showed the absence of any localizing signs.

His memory impairment fully recovered within 24 hours after the onset of symptoms. However, he did not remember the episode, including admission to the hospital and undergoing several examinations. On the day after admission, since recovery of his memory function was confirmed by interview, the patient was discharged on foot. In the three months of follow-up, he has been well without further amnesia episodes.

## Discussion

Our case fully met the Hodges and Warlow TGA diagnostic criteria (4), which include a capable observer witnessing the attack, no neurological symptoms during or after the attack, anterograde amnesia, no personal identity loss, no recent head injury, no history of seizures or active epilepsy, absence of epileptic features, and resolution of TGA within 24 hours. Our patient did not have any head injury or seizure episodes for at least 10 years. We ruled out electrolyte/metabolic abnormality based on normal biochemical test and arterial blood gas analysis results. 

The following differential diagnoses were identified for our patient: complex partial seizures, acute confusional state, transient ischemic attack (TIA), transient epileptic amnesia (TEA), psychogenic amnesia, toxic metabolic states, and migraine. TEA is an indication of temporal lobe epilepsy, and patients will have multiple episodes of amnesia; symptoms commonly last for less than an hour or rapidly recur, which did not happen in our case. Psychogenic amnesia is characterized by profound retrograde amnesia with personal identity loss but intact anterograde memory; these symptoms do not correspond to those of our patient. We ruled out TIA, which manifests additional focal neurological deficits during an attack, as our patient had no motor or sensory loss. Episodic memory dysfunction in TGA presents transiently. In contrast, it may present acutely in concussion, sub-acutely in thiamine deficiency, or chronically in Alzheimer’s disease.

Since the pathophysiology of TGA is obscure, many hypotheses have been proposed. A number of precipitating factors are closely related to TGA, including physical exercise, migraines, sexual intercourse, acute pain, emotional stress, cervical hyperextension, and coughing (5-7). Our patient did not have this type of episode based on information obtained from family members and no factor other than increased blood pressure, can fully explain the occurrence of TGA in our patient. Plausible hypotheses for the pathogenesis of TGA are migraine-like mechanism and cerebral hypoxic-ischemic insult. Studies using neuroimaging technologies like single photon emission CT scan show impaired blood flow in the parahippocampus, hippocampus, and mediobasal temporal region in TGA patients (7). Similarly, diffusion-weighted MRI has demonstrated metabolic stress and structural changes in patients with TGA (8). It has been useful for confirming ischemic amnesia (9). Assessment of internal jugular venous flow has shown blockage and resulting venous ischemia to hippocampal or bilateral diencephalic structures occurring due to Valsalva maneuvers, which may contribute to TGA development (7, 10). The rare incidence of microembolic signals in TGA patients indicates that embolism plays no essential role in TGA development (11).

His anterograde memory rapidly and completely returned within 24 hours, but he was not able to remember the memory loss episode. Like our patient, patients in previous studies have reported that their retrograde memory was slow to recover to normal. 

Arterial hypertension is a prominent finding in patients with TGA and may be an associated risk factor (12). Nedelmann et al. demonstrated that 21 of 22 patients with TGA (60% female, mean age 62.4 years) had high SBP (180.9/98.3 mmHg mean pressure three hours after symptom onset). Moreover, systolic values above 200 mmHg were found in one-third of patients. These clinical observations may suggest that elevated blood pressure is a common factor in the early stage of this condition (3).

## Conclusion:

We encountered the unique case of a patient with a typical TGA history associated with hypertensive emergencies. Our report may help emergency physicians to successfully diagnose the condition by recognizing its characteristic features.

## Other relationships

All authors have declared that they have no other relationships or activities that could appear to have influenced the submitted work.
